# Characterization of the Functional Domain of β2-Microglobulin from the Asian Seabass, *Lates calcarifer*

**DOI:** 10.1371/journal.pone.0013159

**Published:** 2010-10-06

**Authors:** Hirzahida Mohd-Padil, Khairina Tajul-Arifin, Adura Mohd-Adnan

**Affiliations:** School of Biosciences and Biotechnology, Faculty of Science and Technology, Universiti Kebangsaan Malaysia, Bangi, Selangor, Malaysia; Dana-Farber Cancer Institute, United States of America

## Abstract

**Background:**

β2-Microglobulin (β_2_M) is the light chain of major histocompatibility class I (MHC I) that binds non-covalently with the α heavy chain. Both proteins attach to the antigen peptide, presenting a complex to the T cell to be destroyed via the immune mechanism.

**Methodology/Principal Findings:**

In this study, a cDNA sequence encoding β_2_M in the Asian seabass (*Lates calcarifer*) was identified and analyzed using *in silico* approaches to predict and characterize its functional domain. The β_2_M cDNA contains an open reading frame (ORF) of 351 bases with a coding capacity of 116 amino acids. A large portion of the protein consists of the IG constant domain (IGc1), similar to β_2_M sequences from other species studied thus far. Alignment of the IGc1 domains of β_2_M from *L. calcarifer* and other species shows a high degree of overall conservation. Seven amino acids were found to be conserved across taxa whereas conservation between *L. calcarifer* and other fish species was restricted to 14 amino acids at identical conserved positions.

**Conclusion/Significance:**

As the *L. calcarifer* β_2_M protein analyzed in this study contains a functional domain similar to that of β_2_M proteins in other species, it can be postulated that the β_2_M proteins from *L. calcarifer* and other organisms are derived from a common ancestor and thus have a similar immune function. Interestingly, fish β_2_M genes could also be classified according to the ecological habitat of the species, i.e. whether it is from a freshwater, marine or euryhaline environment.

## Introduction

β_2_M is the light chain component of the class I major histocompatibility complex (MHC I) molecule. It consists of about 99 residues with a seven-stranded β-sandwich fold and a central disulfide bond [1] and belongs to the antibody constant domain-like family of proteins (immunoglobulin superfamily). At the cell surface, the MHC I complex is comprised of three extracellular domains from the α chain (α1, α2 and α3) plus the β_2_M protein domain. β_2_M ensures the proper folding and cell surface display of the MHC I molecule [2]. The classical MHC I molecule mainly functions as a component that binds antigenic peptides, presenting them to the T-cell receptor to trigger the cellular immune response [3].

It has been reported that there is a high degree of conservation of β_2_M sequences of mammalian species as well as between mammalian and avian species [4], [5]. The teleost β_2_M sequences also exhibit high sequence similarity overall and conserved regions with warm-blooded vertebrates [6]. Previous phylogenetic analysis revealed the evolutionary diversion of the β_2_M protein in warm-blooded vertebrates and fish [7], [8]. Meanwhile, another earlier phylogenetic analysis indicated that the freshwater fish β_2_M gene diverged from the common ancestor gene earlier than the seawater fish β_2_M gene [6].

In an effort to improve our understanding of the molecular biology of *L. calcarifer*, several thousand expressed sequence tags (ESTs) have been derived from various cDNA libraries from several tissues [9]. Analyses of the EST data enabled us to identify novel gene sequences, including those with significant similarity to β_2_M. Using the numerous β_2_M sequences that are available in the public databases, we analyzed the protein sequence in an effort to better understand the immune system of *L. calcarifer*.

## Materials and Methods

The cDNA sequence of *L. calcarifer* β_2_M obtained from the spleen EST library was translated into its potential open reading frame (ORF) using the ORF Finder algorithm (http://www.ncbi.nlm.nih.gov/gorf/). Domain analyses were carried out using several resources, including Simple Modular Architecture Research Tools (SMART) (http://smart.embl-heidelberg.de/) [10], Pfam 20.0 (http://pfam.wustl.edu/) [11] and Prosite 19.36 (http://www.expasy.org/prosite/) [12]. The profile of the IGc1 domain obtained from the Pfam domain database was used to search for other homologous proteins using the *hmmsearch* program in HMMER version 2.3.2 (http://hmmer.janelia.org) [13], [14] in both the Swiss-Prot database Release 54 (http://www.ncbi.nlm.nih.gov) and the fish genomes at Ensembl database Release 49 (http://www.ensembl.org). The IGc1 domain sequences of the homologous proteins thus identified were extracted for subsequent analyses.

The sequence alignment for the IGc1 domains was built using the *hmmalign* program in the HMMER package against the profile of the IGc1 domain obtained from Pfam to enable the pattern of β_2_M protein change across the taxa to be examined. PHYLIP (http://evolution.genetics.washington.edu/phylip) [15] was then used to perform phylogenetic analyses. A neighbor-joining tree was built using the *protdist* and *neighbor* programs with the Jones-Taylor-Thornton substitution model. The robustness of the trees was evaluated by bootstrap analysis of 1000 random iterations using *seqboot*, while *consense* was used to generate the consensus tree. All programs used to construct the phylogenetic trees are contained in PHYLIP packages [15]. Subsequently, MEGA4 (http://megasoftware.net/) [16] was utilized to view the resultant phylogenetic trees. The *L. calcarifer* β_2_M sequence analyzed in this study has been deposited in the GenBank database under the accession number FJ200516.

## Results

### Analysis of the *L. calcarifer* β_2_M Sequence

Analyses of the cDNA sequence of *L. calcarifer* β_2_M (clone LSE48F06) from the spleen EST library indicated the most probable ORF codes for a polypeptide of 116 amino acids in length. A domain search revealed that a large portion of the protein sequence matched the immunoglobulin C-type (IGc1) domain in the SMART, Pfam and Prosite databases. Almost half of the amino acid residues of β_2_M form two large β structures, which are linked by a central disulfide bond. Its conformation thus strongly resembles the overall tertiary structure of the Igc1 domain [17]. In addition, analysis against the Prosite database showed the presence of an immunoglobulin and major histocompatibility complex protein signature, YSCRVTH, located at residues 97–103 in the *L. calcarifer* β_2_M sequence.

A total of 81 IGc1 domains contained in β_2_M sequences were obtained by protein search against the Swiss-Prot database (version 14 updated 23 October 2007) and the known proteins of five fish species (medaka, stickleback, zebrafish, pufferfish and spotted green pufferfish) in the Ensembl database (version 49 updated March 2008). Of these 81 domain sequences, only 56 were used to build the multiple sequence alignment (MSA) (see Table 1); the remaining sequences were excluded as they were considered identical, truncated or replicated between the two databases used. As the IGc1 domain is the functional domain in all β_2_M sequences analyzed in this study, subsequent analyses focused on this domain as a representation of the β_2_M protein.

**Table 1:** β_2_M sequences used in this analysis.

Group	Protein ID	Protein Name	Species Name	Common Name	Reference
Fish	Q8AXA0	Reg	*R. eglanteria*	Clearnose skate	[19]
	P55076	Bin	*L. intermedius*	Lake Tana barbels	[26]
	O42197	Ipu	*I. punctatus*	Channel catfish	[6]
	Q04475	Dre1	*D. rerio*	Zebrafish	[4]
	Q03422	Cca	*C. carpio*	Common carp	[27]
	Q9PRF8	Aba	*A. baerii*	Siberian sturgeon	[18]
	Q8AYH8	Pol	*P. olivaceus*	Japanese flounder	[8]
	Q90ZJ6	Ola	*O. latipes*	Japanese medaka	[28]
	NP_998291	Dre2	*D. rerio*	zebrafish	[29]
Amphibian	Q9IA97	Xla	*X. laevis*	African clawed frog	[7]
Monotremes	Q864T7	Oan	*O. anatinus*	Platypus	[30]
	Q864T6	Tac	*T. aculeatus*	Australian echidna	[30]
Avians	P21611	Gga	*Gallus gallus*	Chicken	[31]
	P21612	Mga	*M. gallopavo*	Turkey	[32]
Marsupials	Q9GKM2	Tvu	*T. vulpecula*	Brushtail possum	[33]
	Q864T8	Mdo	*M. domestica*	Short-tailed opossum	[30]
Ruminants	Q6QAT4	Oar	*O. aries*	Sheep	Unpublished data
	P01888	Bta	*B. taurus*	Cattle	[34]
Equine	P30441	Eca	*E. caballus*	Horse	[35]
	Q861S3	Eas	*E. asinus*	Ass	[36]
Rodents	P01887	Mmu	*M. musculus*	Mouse	[37]
	P07151	Rno	*R. norvegicus*	Rat	[38]
	Q8CIQ3	Shi	*S. hispidus*	Hispid pocket mouse	Unpublished data
	Q9WV24	Cgr	*C. griseus*	Chinese hamster	[39]
	P01886	Cpo	*C. porcellus*	Domestic guinea pig	[40]
Primates	P55079	Soe	*S. oedipus*	Cotton-top tamarin	[41]
	Q9TSX4	Sfu	*S. fuscicollis*	Brown-headed tamarin	[41]
	O77517	Smn	*S. niger*	Black-handed tamarin	[41]
	O77518	Sim	*S. imperator*	Tamarin	[41]
	P63068	Sbb	*S. bicolor*	Pied bare-faced tamarin	[42]
	O77531	Pir	*P. irrorata*	Bald-faced saki	[41]
	Q6PZD3	Cae	*C.aethiops*	African green monkey	[43]
	O77529	Cho	*C. hoffmannsi*	Hoffmanns's titi	[41]
	O77532	Csa	*C. satanas*	Black-bearded saki	[41]
	Q71UN5	Cpe	*C. penicillata*	Black pencilled marmoset	[42]
	O77533	Cme	*C. melanocephalus*	Black headed Uacari	[42]
	O77519	Lch	*L. chrysopygus*	Golden-rumped lion tamarin	[42]
	O77535	Cpy	*C. pygmaea*	Pygmy marmoset	[42]
	O77530	Cto	*C. torquatus*	Yellow-handed titi	[42]
	O77526	Cpp	*C. personatus*	Masked titi	[42]
	O77528	Cpn	*C. p. nigrifrons*	Black titi	[42]
	O77521	Cem	*C. emiliae*	Emilia's marmoset	[42]
	O77536	Apa	*A. paniscus*	Black spider monkey	[42]
	O77534	Sbo	*S. boliviensis*	Bolivian squirrel monkey	[42]
	O77525	Lla	*L. lagotricha*	Brown woolly monkey	[42]
	O77523	Ase	*A. seniculus*	Howler monkey	[42]
	O77520	Cgo	*C. goeldii*	Goeldi's marmoset	[42]
	P63063	Ale	*A. lemurinus*	Lemurine night monkey	[42]
	O77537	Aaz	*A. azarai*	Azara's night monkey	[42]
	Q6T672	Pan	*P. anubis*	Olive baboon	Unpublished data
	Q8SPW0	Mfa	*M. fascicularis*	Crab-eating macaque	Unpublished data
	P61769	Has	*H. sapiens*	Human	[44]
Others	Q07717	Ssc	*S. scrofa*	Pig	[45]
	P01885	Ocu	*O. cuniculus*	Rabbit	[46]
	Q5MGS7	Fca	*F. catus*	Domestic cat	Unpublished data

The 55 protein sequences used in this study were retrieved from the Swiss-Prot and Refseq databases. Protein ID shows the accession number of the sequence in the database while Protein Name is the protein name designated in this study. Equine, Rodents, Ruminants, Primates and Others are largely grouped as Eutheria.

### Sequence Alignment

**Figure 1:**
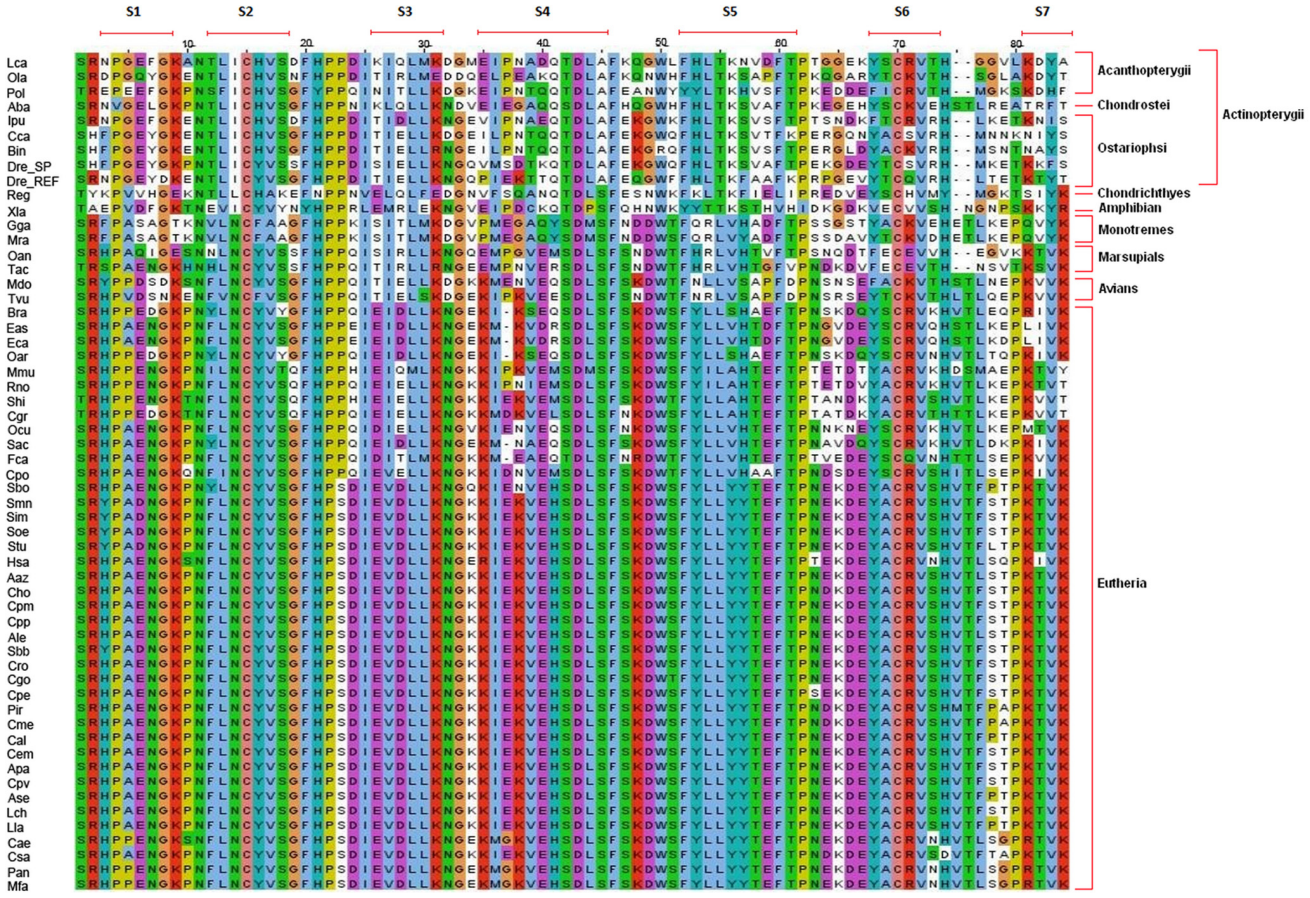
Multiple sequence alignment of IGc1 domains. The alignment consists of IGc1 domain sequences from organisms of various taxa such as Eutheria, Marsupials, Monotremes, Avians, Chondrichthyes fish and Actinopterygii fishes. Lca is the *L. calcarifer* protein sequence and the conserved residues are marked by (*). S1, S2, S3, S4, S5, S6 and S7 indicate the regions of seven β strands in the IGc1 domain. Numbers at the top indicate amino acid positions. Information on the sequences is given in Table 1.

Alignment of the IGc1 domains of β_2_M from *L. calcarifer* and other species showed a high degree of overall conservation across taxa (see Figure 1). The IGc1 domain of *L. calcarifer* starts with a Ser residue, which is conserved among the taxa, with the exception of domains from the Japanese flounder (Q8AYH8), Chinese hamster (Q9WV24), hispid pocket mouse (Q8CIQ3) and Australian echidna (Q864T6), in which the starting residue is a Thr. The fish sequences, with the exception of Siberian sturgeon (Q9PRF8), are two residues shorter (lacking residue-75 and residue-76) than the human (P61769) sequence. The deletions in the teleost sequences are located in the loop between the anti-parallel beta-strands S6 and S7 [18]. Seven positions or residues, Asn_11_, Cys_15_, Pro_22_, Asp_44_, Phe_47_, Cys_70_ and Val_72_ (numbering refers to the position in the MSA) are found to be completely conserved. These residues or regions are believed to be in the active or binding site of the β_2_M protein. A previous study reported that the two cysteine residues (Cys_15_ and Cys_70_), which covalently link to form a disulfide bridge (thus connecting the two β sheets), are important elements in protein folding that contribute to stabilization of the MHC class I molecule [1].

### Phylogenetic Analysis

**Figure 2:**
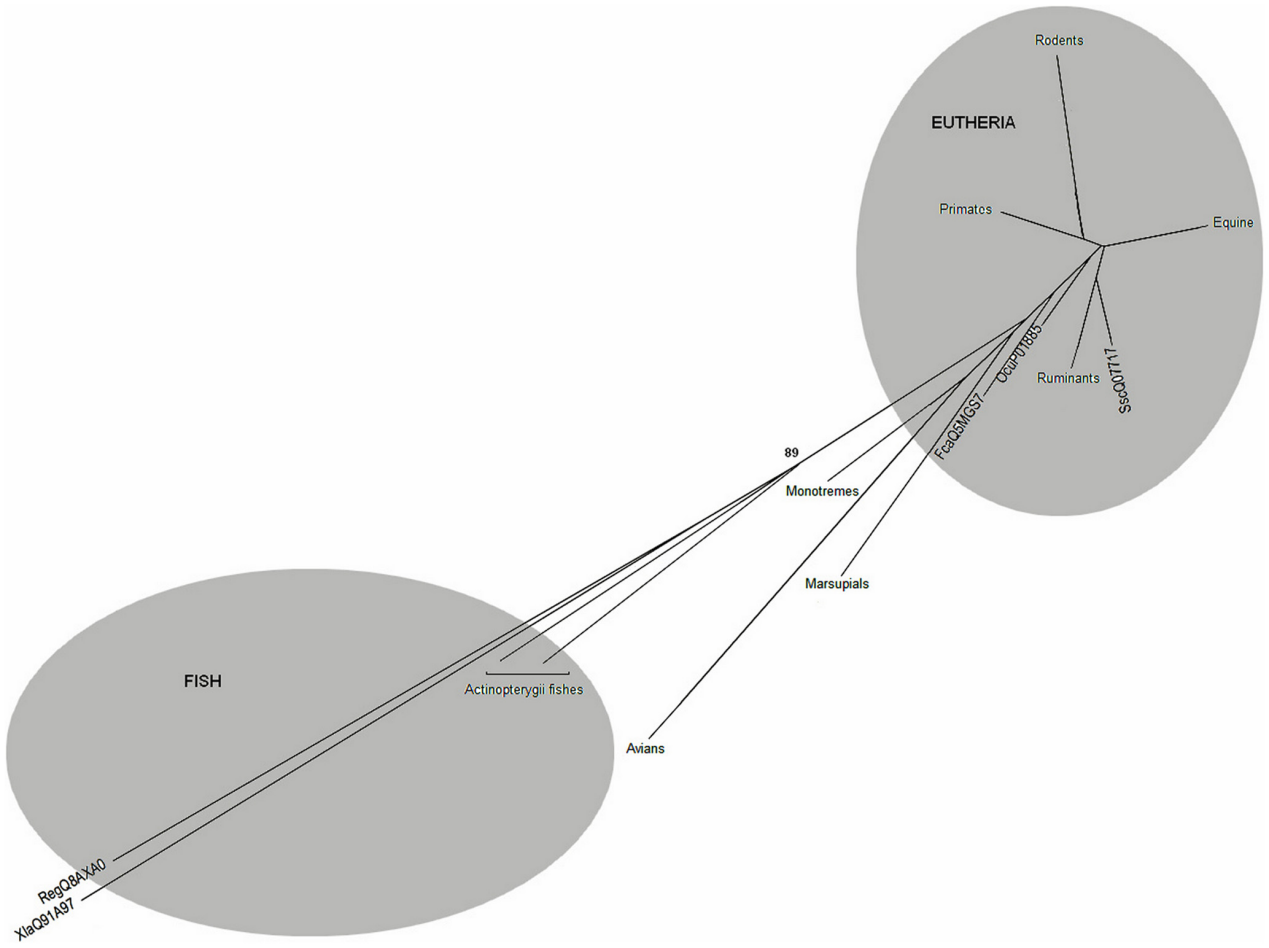
NJ phylogenetic tree of β_2_M protein sequences representing whole organisms. The phylogenetic tree shown is the collapsed tree of 55 sets of sequence data. This tree shows that β_2_M sequences are clustered together according to their taxons. β_2_M sequences from Eutheria are clustered together and consist of sequences from Primates, Equine, Rodents, Ruminants, SscQ07717, OcuP01885 and FcaQ5MGS7. Marsupials, Monotremes and Avians are the intermediate taxons between Eutheria and Fish. Amphibian Xla protein Q9IA97 is clustered together with Actinopterygii fishes while the outgroup in this tree is a cartilaginous fish Reg Q8AXA0. The divergence of fish and mammalian β_2_M received a high bootstrap value (89) to support the reliability of this phylogenetic tree.

**Figure 3:**
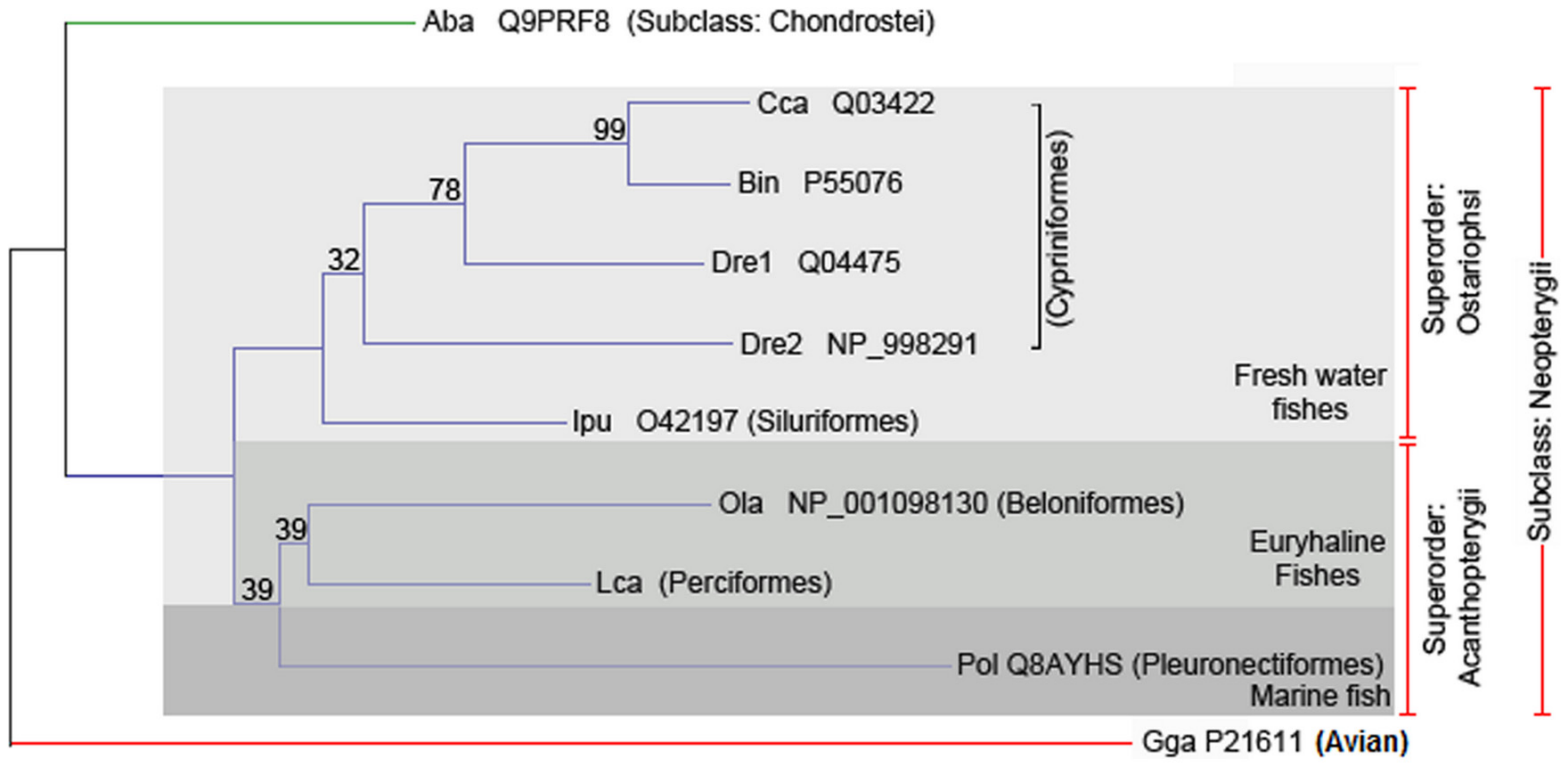
NJ phylogenetic tree of IGc1 domains present in β_2_M protein sequences from fish. The phylogenetic tree shows that fish β_2_M proteins are clustered according to the fish's ecological habitat, which may be fresh water, euryhaline or marine. The chicken sequence was used as the outgroup. Information on the sequences is given in Table 1.

The phylogenetic tree built is an unrooted tree (see Figure 2) with two distinct clades, the mammalian β_2_M and fish β_2_M. The avian, marsupial and monotreme sequences form an intermediate group between the fish and the mammalian clades. All fish sequences are from the Actinopterygii class with the exception of the clearnose skate (Q8AXA0) [19], which is a cartilaginous fish (class Chondrichthyes). In order to clarify the relationships among the β_2_M molecules of the Actinopterygii fish (Siberian sturgeon, channel catfish (O42197), Japanese medaka (Q90ZJ6), Japanese flounder, zebrafish (Q04475, NP_998291), common carp (Q03422) and Lake Tana barbel (P55076)), an NJ tree consisting of fish sequences only was constructed using the chicken (P21611) β_2_M sequence as an outgroup (see Figure 3). The β_2_M from *L. calcarifer,* which is a euryhaline and catadromous species, clustered together with Japanese medaka β_2_M, forming a separate clade from the freshwater fishes (common carp, Lake Tana barbel, zebrafish and channel catfish). The phylogenetic trees also reveal a molecular signal of the ecological distinction between marine and freshwater fish. The Siberian sturgeon β_2_M sequence is the most basal lineage, which is placed outside the main cluster of teleosts in the tree (see Figure 3).

## Discussion

Our analysis of the novel *L. calcarifer* β_2_M gene recovered in this study indicates that, overall, fish β_2_M sequences have high sequence similarity and share many conserved features with published sequences from mammals and birds. Using β_2_M as both a phylogenetic marker and a source of information, we confirmed previous studies indicating that the β_2_M proteins of mammals and fish represent clearly distinct evolutionary paths, with fish β_2_M genes more closely related to avian sequences than to those of mammals [7], [8]. The divergence between fish and mammals is partially a consequence of several unreversed changes in the ORF. For example, at site-14 (S2) in all mammalian β_2_M sequences, the residue is Arg, whereas in Actinopteygii fish β_2_M sequences it is Ile. The Ser residue in mammalian sequences is substituted by Ala at site-45 (S4) in all Actinopterygii fish, whereas Lys at position 55 (S5) in mammalian β_2_M is consistently replaced by Thr in all Actinopterygii fish. Although these changes of amino acids are located within the mature protein region, the residues at those sites are not involved in any important stabilizing interactions of the protein [20].

Our analyses of the Actinopterygii fish β_2_M sequences employed sequences representing two subclasses: Neopterygii (teleosts) and Chondrostei. Within the Neopterygii, two superorders are evident: Ostariophsi and Acanthopterygii. The molecular results showed phylogenetic relationships that support those established based on fish morphology [21]. Ostariophsi includes two different orders (Cypriniformes and Siluriformes), whereas Acanthopterygii includes three different fish orders (Beloniformes, Perciformes and Pleuronectiformes). With the exception of Siberian sturgeon, our results also showed that fishes included in Ostariophsi are mainly freshwater fishes, whereas those of Acanthopterygii are marine or euryhaline fishes (see Figure 3). The results reconfirm a previous paper that indicated an evolutionary divergence had occurred between freshwater and marine or euryhaline fish β_2_M sequences [8]. At the molecular level among the Actinopterygii fishes studied, Asian seabass, Japanese medaka and Japanese flounder sequences share synapomorphic amino acids at three sites: Thr_73,_ (S6) Gly_78_ (loop between S6 and S7) and Asp_82_ (S7). Siberian sturgeon (Chondrost) resolves as the most basal lineage in agreement with other studies [21], [18], confirming this fish is the most primitive member of the subclass Actinopterygii. Indeed, a two-codon (residues 75 and 76 in the alignment) deletion is synapomorphic in all teleost β_2_M sequences in contrast to Siberian sturgeon β_2_M.

Given the preponderance of analytical results and qualitative comparative results, we suggest that the *L. calcarifer* β_2_M gene recovered here is likely to function similarly to previously characterized β_2_M genes, and that the protein it encodes acts as a light chain that binds non-covalently to the heavy chain of the MHC class I molecule. The two proteins would then create a complex with the antigen peptide and present the antigen to T cells to be destroyed by the immune mechanism [22]. β_2_M is also involved in stabilizing the MHC class I molecules [23], [24]. Since β_2_M is clearly most closely related to the IGc1-type domains of MHC class I and II, its gene must have been linked to that of the MHC at some point of evolution [7]. Furthermore, the similarity between the structures of β_2_M and the IGc1-type domains of MHC I and II suggests that they share a common ancestor encoded in MHC genes [25]. In this study, we have identified the β_2_M gene in *L. calcarifer* and confirm its phylogenetic placement within a group of related fish species. We believe that, as more fish β_2_M sequences become available, reanalysis of the data may be able to better resolve the evolutionary history of the seeming ecological divergence detected among fish sequences and that of the *L. calcarifer* β_2_M gene from the rest of the β_2_M gene tree. Again, the overall utility of our approach in the detection, recovery and delineation of genes within *L. calcarifer* is emphasized by its success in our study of β_2_M.
